# Changes of Costimulatory Molecule CD28 on Circulating CD8^+^ T Cells Correlate with Disease Pathogenesis of Chronic Hepatitis B

**DOI:** 10.1155/2014/423181

**Published:** 2014-06-10

**Authors:** Xuefen Li, Haishen Kong, Li Tian, Qiaoyun Zhu, Yiyin Wang, Yuejiao Dong, Qin Ni, Yu Chen

**Affiliations:** ^1^State Key Laboratory for Diagnosis and Treatment of Infectious Diseases, First Affiliated Hospital, School of Medicine, Zhejiang University, 79 Qingchun Road, Hangzhou 310003, China; ^2^Department of Laboratory Medicine, First Affiliated Hospital, School of Medicine, Zhejiang University, 79 Qingchun Road, Hangzhou 310003, China

## Abstract

Costimulatory signals are critical for antiviral immunity. The aim of this study was to evaluate the change of costimulatory molecule CD28 on circulating CD8^+^ T cells in chronic hepatitis B patients (CHB). Seventy CHB patients and fifty-six healthy controls were included, and forty-eight CHB patients were recruited for 52 weeks of longitudinal investigation. The proportions of circulating CD8^+^CD28^+^ and CD8^+^CD28^−^ subpopulations were determined by flow cytometry, and the CD8^+^CD28^+^/CD8^+^CD28^−^ T cells ratio was calculated. Compared with the subpopulation in healthy controls, high proportions of CD8^+^CD28^−^ subpopulation were observed in CHB patients. Similarly, the CD8^+^CD28^+^/CD8^+^CD28^−^ T cells ratio was significantly decreased in CHB patients compared with healthy controls and correlated significantly with hepatitis B virus (HBV) loads. High proportions of CD8^+^CD28^−^ subpopulation and low CD8^+^CD28^+^/CD8^+^CD28^−^ T cells ratio were observed in hepatitis B e antigen- (HBeAg-) positive individuals as compared with that in HBeAg-negative subjects. A significant decrease in CD8^+^CD28^−^ subpopulation, increase in CD8^+^CD28^+^ subpopulation, and CD8^+^CD28^+^/CD8^+^CD28^−^ T cells ratio were seen in those patients who received efficient antiviral therapy. Thus, aberrant CD28 expression on circulating CD8^+^ T cells and the CD8^+^CD28^+^/CD8^+^CD28^−^ T cells ratio reflect the dysregulation of T cell activation and are related to the pathogenesis of chronic HBV infection.

## 1. Introduction

Hepatitis B virus (HBV) is a type of noncytotoxic, hepatotropic DNA virus that causes liver diseases of varying severity. Cellular immunity is critical in determining the outcome of HBV infection [1–3]. In particular, the HBV-specific CD8^+^ T cell response to HBV is thought to play a dominant role in viral clearance and disease pathogenesis [4, 5]. The activation of T cells requires two signals. The first signal is delivered by the binding of an antigen to the MHC molecule and the T cell receptor (TCR). The second signal involves interaction between CD28 on the T cell surface and CD80/86 (a member of the B7 family) on the antigen-presenting cell (APC) [6].

CD28 is known to be the main costimulatory molecule, and it can activate T cells, resulting in proliferation and cytokine secretion [6]. Based on the expression of the costimulatory molecule CD28 on the surface of CD8^+^ T cells, two different lymphocyte subgroups have been designated: antigen-primed cytotoxic T cells (CD8^+^CD28^+^ T cells) and suppressor T cells (CD8^+^CD28^−^ T cells) [6, 7]. The frequency of CD28^+^CD8^+^ T cells and especially the balance between CD8^+^CD28^+^ and CD8^+^CD28^−^ T cells are important in many diseases, including chronic hepatitis B (CHB) [8–11].

Here, we investigated whether expression of the costimulatory molecule CD28 is altered on circulating CD8^+^ T cells and its clinical significance. We assessed the association between the CD28^+^/CD28^−^ ratio in the CD8^+^ T cell population and viremia during pegylated interferon- (Peg-IFN-)*α* therapy. The results provide insight into the effects of antiviral therapy on the balance between CD8^+^CD28^+^ and CD8^+^CD28^−^ T cells and on the outcome of CHB.

## 2. Materials and Methods

### 2.1. Study Subjects

Seventy treatment-naive patients with CHB (52 males and 18 females, aged from 22 to 56) were prospectively enrolled in the study. The diagnosis of chronic HBV infection was made according to the “Guidelines of Chronic Hepatitis B Prevention and Cure,” published by the Society of Hepatology and Infectious Disease, Chinese Medical Association [12]. All patients exhibited the presence of serum hepatitis B surface antigen (HBsAg) on at least two occasions more than 6 months apart, HBV DNA > 3 log_10_ copies/mL, and chronic abnormality in serum alanine aminotransferase (ALT) levels for 6 or more months. Patients coinfected with hepatitis A virus, hepatitis C virus, hepatitis D virus, hepatitis E virus, or human immunodeficiency virus or with other possible causes of chronic liver damage, such as alcohol use, drug use, autoimmune diseases, or congestive heart failure, were excluded from this study. A cohort of age- and sex-matched healthy donors served as controls (*n* = 56) (Table 1). All participating donors gave their informed consent before blood donation. The study protocol, which conformed to the guidelines of the Declaration of Helsinki, was approved by the Ethics Review Committee of the First Affiliated Hospital, School of Medicine, Zhejiang University.

### 2.2. Study Design

Whole-blood samples (2 mL each) were obtained from healthy controls and individual subjects who were receiving Peg-IFN-*α* at five protocol time points (baseline, 12, 24, 36, and 52 weeks). In parallel, routine liver biochemical and virological parameters were detected in serum samples. After 52 weeks of therapy, patients with serum HBV DNA levels that were undetectable by PCR and with normalized serum ALT levels were defined as having received efficient antiviral therapy.

### 2.3. Flow Cytometric Analysis

Freshly anticoagulated peripheral blood (100 *μ*L) was added to flow tubes, and 20 *μ*L of CD3-PerCPCy5.5, CD8-FITC, and CD28-PE mouse anti-human fluorescent monoclonal antibodies (all from Becton Dickinson (BD), Pharmingen, USA) was added. After mixing, the cells were stained for 15 min away from light at room temperature. Red blood cell lysis and cell fixation using a Coulter QPREP specimen processing instrument (Beckman Coulter, FL, USA) were conducted, and more than 1 × 10^4^ lymphocytes were counted using a BD FACSCalibur flow cytometer and CELLQuest software (BD, CA, USA). The results are expressed as the percentages of CD8^+^CD28^+^ and CD8^+^CD28^−^ T cells in the CD3^+^ fraction, and the ratio of CD8^+^CD28^+^/CD8^+^CD28^−^ T cells was calculated.

### 2.4. Serological Liver Function Tests, Virological Assessments, and HBV DNA Detection

Routine serum biochemical parameters, including ALT and aspartate aminotransferase (AST) levels, were measured using automated techniques (HITACHI 7600, Japan) (upper limits of normal: 50 IU/L and 40 IU/L, resp.). HBV markers (HBsAg, hepatitis B e antigen (HBeAg), anti-HBe, and anti-HBc) were detected using a commercial chemiluminescent microparticle immunoassay (CMIA) kit with the Architect i2000 system (Abbott Laboratories, USA).

Serum HBV DNA levels were quantified at all time points using the Cobas HBV Amplicor Monitor assay (Roche Diagnostics, Branchburg, NJ, USA) according to the manufacturer instructions in the reagent kit. The detection limit of the assay was 300 viral genome copies/mL (2.48 log_10_ copies/mL).

### 2.5. Statistical Analysis

GraphPad Prism 5.0 software was used for data processing. The data are expressed as means ± standard errors of the mean (SEM), medians, or percentages. For patients with undetectable HBV DNA levels, the lower limit of detection (2.48 log_10_ copies/mL) was taken as the result for calculation purposes. Comparisons among groups were analyzed using a nonparametric Mann-Whitney rank sum test. Correlation analyses among the detection indicators were conducted using Spearman's correlation coefficient test. Only *P* values below 0.05 were considered to be statistically significant.

## 3. Results

### 3.1. Phenotypic Analysis of CD28 Expression on Circulating CD8^+^ T Cellsin Chronically HBV-Infected Individuals

**Figure 1:**
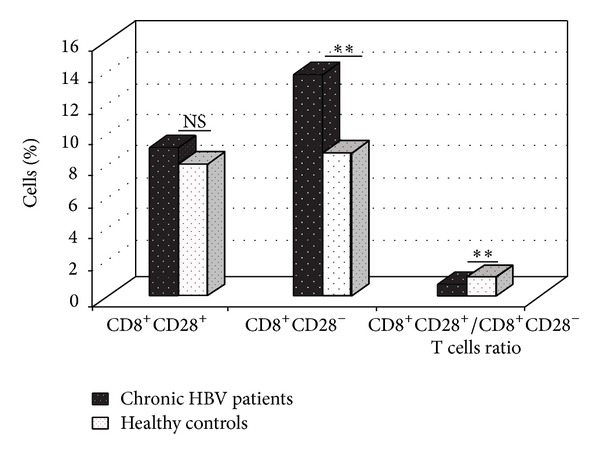
The percentages of CD8^+^CD28^+^ and CD8^+^CD28^−^ subpopulations in the peripheral blood of chronic HBV patients (*n* = 70) and healthy controls (*n* = 56) were analyzed on a flow cytometer, and the CD28^+^/CD28^−^ ratios in the CD8^+^ T cell population were calculated. Patients had higher fractions of CD28^−^ cells and lower ratios of CD28^+^/CD28^−^ in the CD8^+^ T cell population than did healthy controls. NS: no significant difference. **P < 0.01 (unpaired* t*-test).

The proportions of CD8^+^CD28^+^ and CD8^+^CD28^−^ T cells in the peripheral blood of chronic HBV patients (*n* = 70) and healthy controls (*n* = 56) were investigated. High proportions of CD8^+^CD28^−^ T cells were observed in chronically HBV-infected patients compared with healthy controls (*P* < 0.01). A reverse variation in the CD8^+^CD28^+^/CD8^+^CD28^−^ T cells ratio between chronic HBV patients and healthy controls was observed (*P* < 0.01). However, no significant differences were detected in the proportion of CD8^+^CD28^+^ T cells in the CD8^+^ population between healthy controls and HBV-infected patients (Figure 1).

### 3.2. Patterns of CD8^+^CD28^+^ and CD8^+^CD28^−^ T Cells and CD28^+^/CD28^−^ Ratio among CD8^+^ T Cellsin HBeAg-Negative and HBeAg-Positive Patients

**Figure 2:**
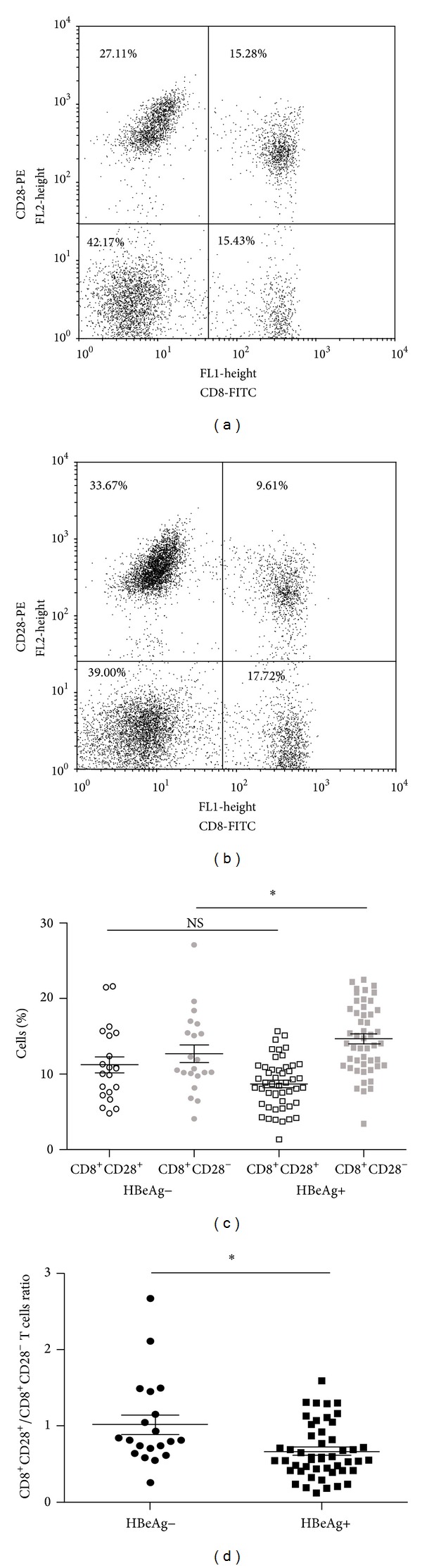
CD28 expression on the CD8^+^ T cells in chronic HBV patients who were HBeAg-positive or HBeAg-negative. The percentages of CD8^+^CD28^+^ and CD8^+^CD28^−^ T cells in (a) HBeAg-negative patient and (b) HBeAg-positive patient. (c) The median values of CD8^+^CD28^+^ and CD8^+^CD28^−^ T cells and (d) the CD28^+^/CD28^−^ ratio in the CD8^+^ T cell population are represented. NS: no significant difference. *P < 0.05 (unpaired* t*-test).

Among the 70 chronically HBV-infected patients, 49 were HBeAg-positive and 21 were HBeAg-negative (Table 1). To further characterize the intensity of CD28 expression on the CD8^+^ T cells, we compared the proportions of CD8^+^CD28^+^ and CD8^+^CD28^−^ T cells and CD28^+^/CD28^−^ ratio in the CD8^+^ T cell population between HBeAg-negative and HBeAg-positive patients. The medians of CD8^+^CD28^+^, CD8^+^CD28^−^ subpopulations, and CD8^+^CD28^+^/CD8^+^CD28^−^ T cells ratio in the HBeAg-negative and HBeAg-positive individuals were 11.23* versus *8.69, 12.66* versus* 14.66, and 1.01* versus* 0.67, respectively, with a significant difference observed in CD8^+^CD28^−^ T cells and CD28^+^/CD28^−^ ratio between these groups (*P* < 0.05) (Figure 2).

### 3.3. Dynamic Changes of CD28 Expression and the CD28^+^/CD28^−^ Ratio in the CD8^+^ T Cell Population during Efficient Antiviral Therapy

**Figure 3:**
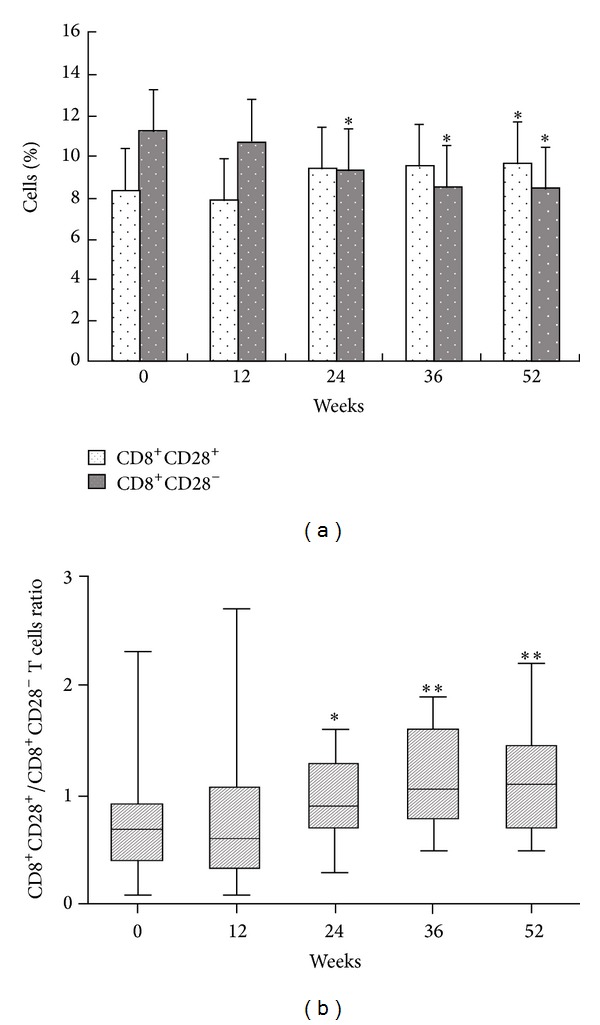
Longitudinal analysis of peripheral blood CD8^+^CD28^+^ and CD8^+^CD28^−^ T cells (a) and CD8^+^CD28^+^/CD8^+^CD28^−^ T cells ratio (b) in 48 CHB patients over a 52-week course of Peg-IFN-*α* therapy. *P < 0.05, **P < 0.01, for the difference in the peripheral blood CD8^+^CD28^+^, CD8^+^CD28^−^ T cell distribution and CD8^+^CD28^+^/CD8^+^CD28^−^ T cells ratio between baseline and week 12, 24, 36, or 52.

CD28 on circulating CD8^+^ T cells and CD28^+^/CD28^−^ ratio in the CD8^+^ T cell population was analyzed in 48 patients with serum HBV DNA levels that were undetectable by PCR and with normalized serum ALT levels at week 52. There were no changes during the early phase (≤12 weeks). Subsequently, the proportions of CD8^+^CD28^−^ T cells decreased and CD8^+^CD28^+^/CD8^+^CD28^−^ T cells ratios increased gradually and achieved a more significant difference than that at baseline (*P* < 0.05) (Figures 3(a) and 3(b)). The level of CD8^+^CD28^+^ T cells achieved a highly significant difference at week 52 in comparison to the level observed at baseline (*P* < 0.05) (Figure 3(a)).

### 3.4. The CD28^+^/CD28^−^ Ratio among CD8^+^ T Cells in relation to the HBV DNA Load and the ALT Level

**Figure 4:**
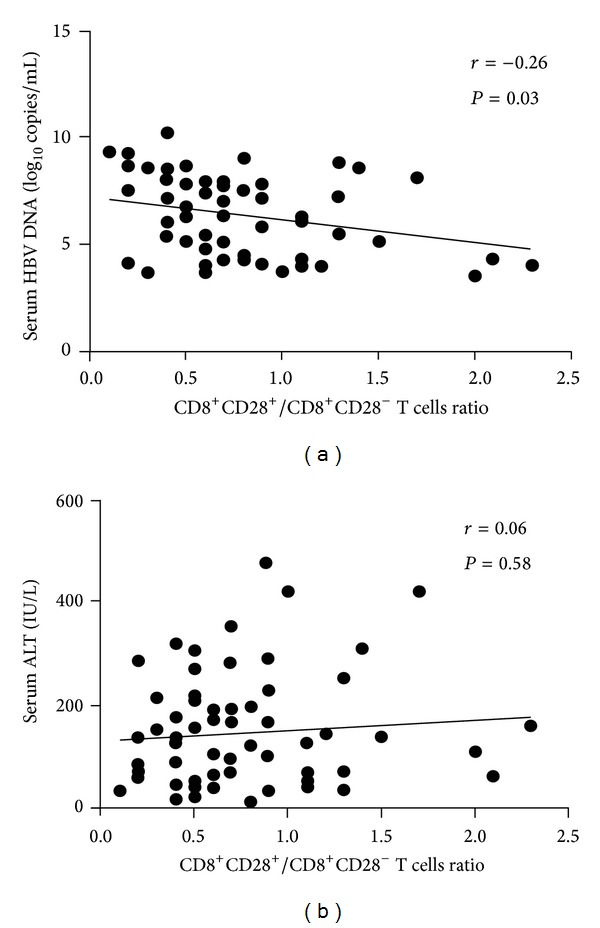
Correlations of CD28^+^/CD28^−^ ratios in the CD8^+^ T cell population with (a) HBV DNA loads (*n* = 70) and (b) serum ALT levels (*n* = 70).* r* and *P* values are shown.

We next analyzed the correlation of the CD28^+^/CD28^−^ ratio in the CD8^+^ T cell population with HBV DNA loads and ALT levels in chronically infected, untreated patients. The CD8^+^CD28^+^/CD8^+^CD28^−^ T cells ratio in HBV-infected patients was inversely correlated with the viral load (*n* = 48, *P* < 0.05) (Figure 4(a)). In particular, patients with higher HBV DNA loads had lower CD8^+^CD28^+^/CD8^+^CD28^−^ T cells ratios in their peripheral blood. No correlation was identified between the CD8^+^CD28^+^/CD8^+^CD28^−^ T cells ratio and serum ALT levels (Figure 4(b)).

## 4. Discussion

There are approximately 400 million HBV carriers around the world, 15% of whom develop CHB every year and eventually progress to liver cirrhosis and hepatocellular carcinoma [13]. The host immune response plays a vital role in the pathogenesis and clinical outcomes of hepatitis B. Disorder of the immune system and especially disorder of CD8^+^ T-cell-mediated immunity are major reasons that the virus cannot be eliminated; instead, it persists indefinitely [2, 3, 14].

CD28 is an important molecular marker that is expressed on T cell surfaces and plays an important role in the activation of the T-cell-mediated immune response [6, 15]. A decrease in CD28 expression may lead to T cell dysfunction. CD8^+^ T cells that also express CD28 (CD8^+^CD28^+^ T cells) participate in virus clearance. In contrast, CD8^+^ T cells that do not express CD28 (CD8^+^CD28^−^ T cells) have an immune inhibition function and may suppress the antigen-presenting function of APCs and may thus indirectly inhibit the activation and amplification of T cells [6, 16]. CD8^+^CD28^+^ and CD8^+^CD28^−^ T cells have opposite effects on virus removal; thus, the balance of CD8^+^CD28^+^ and CD8^+^CD28^−^ T cells and especially that of CD8^+^CD28^+^/CD8^+^CD28^−^ T cells ratio can reflect the immune status of CD8^+^ T cells.

In this study, we evaluated the expression of costimulatory molecule CD28 on circulating CD8^+^ T cells and the balance of CD8^+^CD28^+^ and CD8^+^CD28^−^ T cells in patients with hepatitis B. Compared with healthy controls, high level of CD8^+^CD28^−^ subpopulations and low CD28^+^/CD28^−^ ratio in the CD8^+^ T cell population were observed in patients with hepatitis B. Similar variations in CD8^+^CD28^−^ T cells proportions and CD8^+^CD28^+^/CD8^+^CD28^−^ T cells ratio were also observed between HBeAg-positive group and HBeAg-negative group. Further study revealed that the CD28^+^/CD28^−^ ratio in the CD8^+^ T cell population was significantly related to HBV DNA loads but not to biological indicators of liver function (e.g., ALT levels). Our data also showed that CD8^+^CD28^+^ T cells proportions and CD8^+^CD28^+^/CD8^+^CD28^−^ T cells ratio were enhanced and the levels of CD8^+^CD28^−^ T cells declined in chronic HBV patients given effective therapy with Peg-IFN-*α*. Therefore, the expression of the costimulatory molecule CD28 on circulating CD8^+^ T cells might be a vital reason for the inactive T cells, resulting in an incapacity to eliminate the virus.

When an individual is exposed to HBV, the strength of the immune response, especially the HBV-specific cytotoxic T lymphocytes (CTLs), plays an important role in viral control and liver damage [2, 3]. Studies by Webster Bertoletti showed that expression of costimulatory molecule CD28 on circulating CD8^+^ T cells can promote those T cell subsets differentiating into specific CTLs during stimulation with an antigen and play a critical role in virus clearance [17]. The percentage of CD8^+^CD28^−^ T cells increases in patients with CHB, and CD8^+^ T cells that do not express CD28 cannot differentiate into specific CTLs during antigen stimulation, resulting in failure to clear the virus. The phenotype of CD8^+^CD28^+^ T cells might become altered during antigen stimulation in vitro, with a subset converting to CD8^+^CD28^−^ T cells [18]. The phenotype of CD8^+^CD28^+^ T cells in patients with CHB could also be altered during long-term HBV antigen stimulation, with a constant transformation of CD8^+^CD28^+^ T cells into CD8^+^CD28^−^ T cells. CD8^+^CD28^−^ T cells, which have an immune inhibition function, can repress antibody production and CTL activity. The increase in CD8^+^CD28^−^ T cell numbers makes it more difficult to clear HBV infection.

In conclusion, our study suggests that the imbalance of CD8^+^CD28^+^ and CD8^+^CD28^−^ T cells is associated with disease pathogenesis in patients with CHB. The balance of CD8^+^CD28^+^ and CD8^+^CD28^−^ T cells is important in CHB, and tilting the balance toward CD8^+^CD28^+^ T lymphocytes is beneficial for CHB patients.

## Figures and Tables

**Table 1:** Patient and control characteristics.

	Chronic HBV patients (*n* = 70)	Controls (*n* = 56)
Male, *n* (%)	52 (74.3%)	48 (85.7%)
Age (years)*	33.7 ± 1.1	39.8 ± 1.8
Serum ALT (IU/L)*	155.7 ± 14.1	15.4 ± 1.6
HBV DNA (log_10_ copies/mL)*	6.6 ± 0.3	Neg.
HBeAg-positive	49 (70%)	n.a.

ALT, alanine aminotransferase; n.a., not applicable; Neg., negative.

*Means ± standard error of the mean (SEM).
